# Comparative evaluation of the sexual functions and NF-κB and Nrf2 pathways of some aphrodisiac herbal extracts in male rats

**DOI:** 10.1186/s12906-016-1303-x

**Published:** 2016-08-26

**Authors:** Kazim Sahin, Cemal Orhan, Fatih Akdemir, Mehmet Tuzcu, Hasan Gencoglu, Nurhan Sahin, Gaffari Turk, Ismet Yilmaz, Ibrahim H. Ozercan, Vijaya Juturu

**Affiliations:** 1 Department of Animal Nutrition, Faculty of Veterinary Science, Firat University, 23119 Elazig, Turkey; 2 Department of Nutrition, Faculty of Fisheries, Inonu University, Malatya, 44280 Turkey; 3 Division of Biology, Faculty of Science, Firat University, 23119 Elazig, Turkey; 4 Department of Reproduction and Artificial Insemination, Faculty of Veterinary, Firat University, 23119 Elazig, Turkey; 5 Department of Pharmacology, Faculty of Pharmacy, Inonu University, Malatya, 44100 Turkey; 6 Department of Pathology, Faculty of Medicine, Firat University, 23119 Elazig, Turkey; 7 Research and Development, OmniActive Health Technologies Inc., Morristown, NJ USA

**Keywords:** *Mucuna pruriens*, *Tribulus terrestris*, *Withania somnifera*, Sexual enhancer, Fertility, Reproductive organs

## Abstract

**Background:**

*Mucuna pruriens*, *Tribulus terrestris and* Ashwagandha (*Withania somnifera*) are widely known as antioxidant effective herbals and have been reported to possess aphrodisiac activities in traditional usages. In this study, we determined the effects of these herbals on sexual functions, serum biochemical parameters, oxidative stress and levels of NF-κB, Nrf2, and HO-1 in reproductive tissues.

**Methods:**

Thirty-five male rats were divided into five groups: the control group, sildenafil-treated group (5 mg/kg/d), Mucuna, Tribulus and Ashwagandha groups. The extract groups were treated orally either with Mucuna, Tribulus or Ashwagandha (300 mg/kg b.w.) for 8 weeks.

**Results:**

All of the extracts were found to be significantly effective in sexual functioning and antioxidant capacity and Tribulus showed the highest effectiveness. Serum testosterone levels significantly increased in Tribulus and Ashwagandha groups in comparison to control group. Tribulus was able to reduce the levels of NF-κB and increase the levels of Nrf2 and HO-1 to a much greater extent than Mucuna and Ashwagandha.

**Conclusions:**

These results demonstrate for the first time that Mucuna, Tribulus and Ashwagandha supplementation improves sexual function in male rats via activating Nrf2/ HO-1 pathway while inhibiting the NF-κB levels. Moreover, *Tribulus terrestris* extract was found to be more bioavailable from Ashwagandha extract followed by Mucuna extract.

**Graphical abstract:**

Schematic representation of the mode of action of some aphrodisiac herbal extracts to improve sexual functions
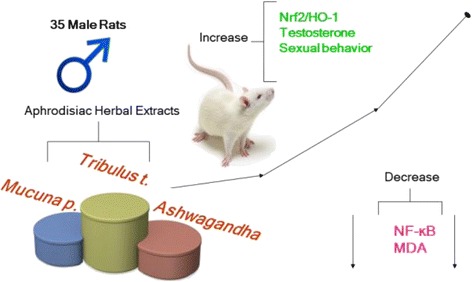

## Background

Male infertility and reproductive dysfunctions are serious widespread health problems and almost half of the human infertility is considered to be male, moreover, the etiology is not obvious in 40 to 50 % of infertile males as well [1, 2]. It has also been observed that oligospermia is the single most common reason for the depressed male fertility [3]. Metabolic generation of reactive oxygen species (ROS) is required for male sexual function, whilst high levels of ROS may be the reason for low sperm quality and male infertility [4].

Animal experiments suggest that a better treatment for sexual dysfunction or infertility may not only improve sexual relationships but also the overall quality of life [5]. Free radicals are extremely reactive molecules which include reactive oxygen species (ROS) and also nitrogen species. These radicals are normally generated by subcellular compartments of testes, particularly mitochondria; however excessive free radical production can cause tissue injury and cell death and result as depleting the antioxidant status [6]. In many countries, different varieties of plants have been used as sexual stimulants in traditional medicine including *Mucuna pruriens*, *Tribulus terrestris*, and Ashwagandha [7–9]. *Mucuna pruriens* (commonly known as Cowitch), a leguminous plant grown in Africa, South America, and South Asia is identified as an herbal medicine for improving the antioxidant, antidiabetic, antidiabetes-induced erectile dysfunction, anti-inflammatory, neuroprotective, aphrodisiac, anticataleptic and antiepileptic, antibacterial, cardioprotective properties [10–13]. *Tribulus terrestris* (belonging to family Zygophyllaceae), known as Gokshur or Gokharu, has been used for a long time for treatment of various kinds of diseases by anti-inflammatory, antidiabetic, hypolipidemic, cardiotonic, hepatoprotective, analgesic, antispasmodic, anticancer, and antibacterial activities [14, 15]. *Withania somnifera* (Ashwagandha) is one of the most valuable herbs in the traditional Indian systems of medicine [16] and it possesses immunomodulatory, anticancer, antimetastatic, antistress, antioxidant, hemopoietic, rejuvenating properties and besides its positive influence on the endocrine, cardiopulmonary and central nervous system [17, 18]. Additionally, *Mucuna pruriens* also is shown to improve sperm density, motility, serum testosterone levels, male sexual behavior and even androgenic in hyperglycemic male rats [19, 20]. *Tribulus terrestris* has the potential to increase hormone levels of testosterone and enhance premature ejaculation [21, 22]. Ashwagandha has the capacity to improve blood circulation in the body, thus naturally enhance sperm quality [8, 23].

Diminishing the excessive ROS levels is suggested for being a penetrating way to overcome infertility problems during the aging [24] and even enhancing the testosterone alone were shown to reduce ROS production and increase p-eNOS/eNOS ratio in the castrated rats, therefore, ameliorate erectile dysfunction as well [25]. The signaling pathway of erythroid 2-related factor 2 (Nrf2) antioxidant response element (ARE) plays a key role in oxidative stress response and Nrf2 was suggested to be a transcription factor that promotes the expression of many crucial antioxidant genes [26, 27]. Heme oxygenase (HO) pathway has a major role in male reproductive system and sexual dysfunctions and heat shock protein 32 or heme oxygenase-1 protein (HO-1) is known to be an inducible isoform protein and it is induced by many different conditions and agents including hypoxia, cytokines, oxidative stress, heat shock, and reactive oxygen species [28, 29]. The nuclear factor kappa-B (NF-κB), is an enhancer-binding transcription factor located in the immune response and participates to cell proliferation and apoptosis, meanwhile, NF-κB binds to DNA at 50 kDa subunit (p50) and involves the transcriptional activation with the 65 kDa subunit (p65) [30]. The molecular mechanism including NF-κB and Nrf2 pathway of *Tribulus terrestris*, *Mucuna pruriens* and Ashwagandha extracts as an antioxidant in reproductive tissues of male rats has not been investigated. Based on the current knowledge, the aim of the present study was to determine the effects of the *Tribulus terrestris*, *Mucuna pruriens* and Ashwagandha on levels of serum biochemical parameters, hormones, MDA (malondialdehyde) and the expression of Nrf2 and NF-κB in reproductive tissues of male rats.

## Methods

### Animals

Thirty-five Sprague-Dawley male rats (age: 8 weeks, weight: 180 ± 20 g) were provided by the Laboratory of Experimental Animals of Inonu University, Malatya, Turkey and the Animal Care Committee of the Inonu University approved this study (2014/A-53). The rats were housed in standard plastic cages (10.25″w × 18.75″1 × 4″d), in a controlled environment with humidity (55 ± 5 %) under a 12:12-h light-dark cycle at 22 °C. Rats were provided with standard diet and tap water ad libitum. The animals were housed in plastic wire meshed cages in the animal house. The wooden material used as bedding was replaced every 3 days. All the experiments were conducted between 09.30 and 17.00 h to minimize the effects of environmental changes.

### Experimental design

The rats were housed in plastic metabolic cages and were randomly divided into 5 groups of seven. These groups were as follows: (i) Group I – Negative control: Rats only fed with standard diet and tap water as vehicle, (ii) Group II – Positive control: Rats were fed with standard diet and treated with sildenafil citrate (Viagra, Pfizer) orally via an intragastric tube (5 mg/kg/d), (iii) Group III: Rats were fed with standard diet and treated with *Mucuna pruriens* (iv) Group IV: Rats were fed with standard diet treated with *Tribulus terrestris* Group V: Rats were fed with standard diet and treated with Ashwagandha.

The animals were treated with dried seed powder of *Mucuna pruriens* extracted with an ethanol-water mixture (70:30) (Item code: 51002; active ingredient: min.20 % L-DOPA), dried fruit extract powder of *Tribulus terrestris* extracted with a methanol-water mixture (70:30) (Item code: 35002; active ingredient: min. 40 % saponis) and dried roots of the plant *Withania somnifera* extracted with a methanol-water mixture (70:30) (Item code: 13002; active ingredients: min: 2.5 % total withanoloids). The purity of all the extracts was 98 %. All the extracts were dissolved in distilled water and administered orally via an intragastric tube daily with an optimal dose of 300 mg/kg body weight for 8 weeks in parallel with sildenafil citrate treated positive control group which was also administered in the same way like the other extract treated groups based on preliminary and published data [31]. All of the herbal extracts were supplied by Omni Active Health Technologies Pvt. Ltd. (Mumbai, India).

Animals were sacrificed under the ether anesthesia and the testes, epididymis, vas deferens, and ventral prostate were removed and cleared from adhering connective tissue and weighed. One of the testis samples was fixed in Bouin’s solution for histopathological examination. The other testes samples were stored at 20 ° C for biochemical analyses. Testes were taken from 20 °C freezer and immediately transferred to the cold glass tubes. Then, the testes were diluted with a nine-fold volume of phosphate-buffered saline (PBS; pH 7.4). For the enzymatic analyses, testes were minced in a glass and homogenized by a Teflon–glass homogenizer for 3 min in cold physiological saline on ice [32]. The cauda epididymidis was cut longitudinally with a pair of fine-pointed scissors and compressed with forceps. The sperm was released by mincing the cauda epididymis into pieces on the Petri dishes that contained phosphate buffer saline (PBS) for sperm characteristics analysis. Since epididymis came in pairs, one cauda epididymis was put in a Petri dish containing 10 ml of 0.1 M PBS specifically for sperm count and sperm motility analysis while the other cauda epididymis put in another Petri dish containing 1 ml 0.1 M PBS for sperm viability and sperm morphology. The spermatozoa were allowed to flow out from cauda epididymis into the buffer. Then, the sperm suspensions were left at room temperature for 10 min for the suspension to allow sperm to swim out of the lumen of the cauda epididymidis for sperm characteristics analysis.

### Sperm quality

Sperm analyses were performed using the methods previously reported in the study by Turk et al. [33]. Sperm count was determined using the hemocytometer under a light microscope. A cover slip was placed on the hemocytometer before a drop with 10 μl of caudal epididymal sperm solution loaded under the cover slip. Haemocytometer was again used for sperm motility analysis. A cover slip was placed on the hemocytometer before a drop with 10 μL of caudal epididymal sperm solution was loaded under the cover slip. Sperm viability analysis will be used the sperm from the other cauda epididymis that will be put in a Petri dish with 1 ml 0.1 M PBS. On a clean glass slide, 1 drop of sperm suspension will be gently mixed with 3 drops of eosin using the sharp glass slide end. After 30 s, 1 drop of nigrosin was mixed together with the solution and a smear will be made. The smear will be then air-dried and observed under x200 magnification of imaging microscope. The sperm will be counted based on the degree of membrane permeability. The dead sperm will be showed pink coloration of the head whereas the viable sperm will be showed whitish or colorless head. Sperm morphology analysis will be used the same sperm smear made for sperm viability analysis. This time, the sperm will be observed under x400 magnification of imaging microscope to clearly evaluate the morphology of the sperm head, neck, and tail. The sperm will be generally classified as normal or abnormal without further characterized the types of abnormality found on the sperm. The normal sperm will be given a score of 100 and the abnormal one will be given a score of “0” to enable statistical analysis by using Statistical Analysis System (SAS) to be carried out easily.

### Laboratory analyses

Hematology parameters in whole blood samples were analyzed by automated analyzer Exigo EOS Vet (Boule Medical AB, Spanga, Sweden). Serum aspartate aminotransferase (AST), alanine aminotransferase (ALT), and alkaline phosphatase (ALP), creatine kinase (CK) concentrations were analyzed by the biochemical analyzer (Samsung LABGEO-PT10). Serum testosterone, luteinizing hormone (LH), follicle stimulating hormone (FSH) levels were measured by using an ELISA kit (Cayman Chemical Company, Ann Arbor, Michigan, USA; Elx-800; Bio-Tek Instruments Inc, Vermont, USA).

A 10 % (w/v) tissue homogenate was prepared in 10 mM phosphate buffer (pH 7.4). The homogenate was centrifuged at 13,000 g for 10 min at 4 °C. The supernatant was collected and stored at -80 °C. The concentration of MDA, an index of lipid peroxidation and oxidative stress, was measured using the fully automatic HPLC (Shimadzu, Kyoto, Japan) equipped with a pump (LC-20 AD), an ultraviolet-visible detector (SPD-20A), an inertsil ODS-3 C18 column (250 × 4.6 mm, 5 m), a column oven (CTO-10ASVP), an autosampler (SIL-20A), a degasser unit (DGU-20A5) and a computer system with LC solution Software (Shimadzu) [34].

### Western blot analysis for NF-κB p65, Nrf-2 and HO-1

Protein (NF-κB, Nrf2, and HO-1) levels were analyzed by western blotting technique. For western blotting; epididymis, prostate, testes and vas deferens tissues of the rats were removed after sacrification to analyze the target protein expressions among the groups. Briefly, accurately weighed each tissue sample was homogenized in 1:10 (w/v) in 10 mM Tris-HCl buffer at pH 7.4, containing 0.1 mM NaCl, 0.1 mM phenylmethylsulfonyl fluoride, and 5 μM soybean (soluble powder; Sigma, St. Louis, MO, USA) as trypsin inhibitor. Tissue homogenate was centrifuged at 15,000 *g* at 4 °C for 30 min, and the supernatant was transferred into fresh tubes. Sodium dodecyl sulfate-polyacrylamide gel electrophoresis sample buffer containing 2 % *β*-mercaptoethanol was added to the supernatant. Equal amounts of protein (20 μg) were electrophoresed and subsequently transferred to nitrocellulose membrane (Schleicher and Schuell Inc., Keene, NH, USA). Nitrocellulose blots were washed twice for 5 min in phosphate buffered saline (PBS) and blocked with 1 % bovine serum albumin in PBS for 1 h prior to the application of primary antibody. Rat antibodies against NF-κB 65, Nrf-2 and HO-1 were purchased from Abcam (Cambridge, UK). Primary antibody was diluted (1:1000) in the same buffer containing 0.05 % Tween-20. The nitrocellulose membrane was incubated overnight at 4 °C with protein antibody. The blots were washed and incubated with horseradish peroxidase-conjugated goat anti-mouse IgG (Abcam, Cambridge, UK). Specific binding was detected using diaminobenzidine and hydrogen peroxide as substrates. Protein loading was controlled using a monoclonal mouse antibody against β-actin antibody (A5316; Sigma). Band intensities of the proteins were quantified by densitometric analysis using an image analysis system (Image J; National Institute of Health, Bethesda, USA). Samples were analyzed in quadruplicate, and a representative blot is shown in the respective figures. Results were normalized to the β-actin expression in each group as percent of control.

### Statistical analysis

Data analysis was performed using Statistical Analysis System (SAS) version 9.2. Data of body weight, serum testosterone, mounting latency and mounting frequency were subjected to analysis of variance (ANOVA) to analyze the significant treatment effect and the mean between the groups was compared using Duncan Multiple Range Test if F value was significant at *P* < 0.05.

## Results

No significant change of the extracts was observed on final body weight, absolute and relative reproductive organ weights of the animals among the groups (*P* > 0.05) (Table 1). Sexual behavior changes were presented in Table 2. All the treatment groups showed significant decreases in mounting latency and intromission latency values (*P* < 0.0001), and significant increases in mounting frequency and intromission frequency values when compared to the standard control group (*P* < 0.0001).

**Table 1:** The effects of extracts on final body weight, absolute and relative reproductive organ weights

Item	Groups					--*P*--
	Control	Sildenafil	*Mucuna pruriens*	*Tribulus terrestris*	*Ashwagandha*	
Final body weight, g	324.00 ± 24.84	316.57 ± 17.99	312.00 ± 29.73	307.71 ± 52.45	331.80 ± 28.31	>0.05
Testis, g	1.39 ± 0.07	1.35 ± 0.11	1.35 ± 0.09	1.36 ± 0.14	1.33 ± 0.08	>0.05
Whole epididymis, g	0.58 ± 0.05	0.61 ± 0.04	0.56 ± 0.05	0.58 ± 0.07	0.59 ± 0.04	>0.05
Right cauda epididymis, g	0.25 ± 0.03	0.24 ± 0.03	0.24 ± 0.03	0.23 ± 0.05	0.23 ± 0.02	>0.05
Vas deferens, g	0.12 ± 0.01	0.13 ± 0.01	0.12 ± 0.01	0.13 ± 0.01	0.13 ± 0.01	>0.05
Seminal vesicles, g	1.16 ± 0.30	1.10 ± 0.25	1.09 ± 0.18	1.17 ± 0.24	1.39 ± 0.31	>0.05
Ventral prostate, g	0.43 ± 0.07	0.49 ± 0.07	0.41 ± 0.07	0.50 ± 0.17	0.48 ± 0.18	>0.05
Testis*, %	0.43 ± 0.04	0.43 ± 0.04	0.43 ± 0.05	0.45 ± 0.05	0.40 ± 0.03	>0.05
Whole epididymis*, %	0.18 ± 0.02	0.19 ± 0.02	0.18 ± 0.02	0.19 ± 0.02	0.18 ± 0.01	>0.05
Right cauda epididymis*, %	0.08 ± 0.01	0.08 ± 0.01	0.08 ± 0.01	0.08 ± 0.01	0.07 ± 0.004	>0.05
Vas deferens*, %	0.04 ± 0.005	0.04 ± 0.008	0.04 ± 0.003	0.04 ± 0.007	0.04 ± 0.004	>0.05
Seminal vesicles*, %	0.36 ± 0.09	0.35 ± 0.09	0.35 ± 0.05	0.38 ± 0.06	0.42 ± 0.9	>0.05
Ventral prostate*, %	0.13 ± 0.02	0.15 ± 0.02	0.13 ± 0.02	0.16 ± 0.04	0.14 ± 0.05	>0.05

*Relative reproductive organ weights [organ weight (g) / final body weight (g) X 100]

Control, no treatment; Sildenafil, rats treated with Sildenafil (5 mg/kg/d); Mucuna, rats treated with Mucuna pruriens (300 mg/kg bw); Tribulus; rats treated with Tribulus terrestris (300 mg/kg bw); Ashwagandha; rats treated with Ashwagandha (300 mg/kg bw). Data are LS means ± SE (*n* = 7). Different superscripts in the same row (a–c) indicate group mean differences (*p* < 0.05)

**Table 2:** The effects of extracts on sexual behaviors in rats

Item	Groups					--*P*--
	Control	Sildenafil	*Mucuna pruriens*	*Tribulus terrestris*	*Ashwagandha*	
Mounting Latency, sec	9.48 ± 1.20^a^	2.10 ± 0.34^d^	5.27 ± 0.62^b^	2.86 ± 0.62^cd^	3.15 ± 0.60^c^	0.0001
Mounting Frequency*	65.65 ± 8.19^d^	214.27 ± 17.76^a^	118.99 ± 7.51^c^	170.86 ± 10.42^b^	167.72 ± 14.91^b^	0.0001
Intromission Latency, sec	9.40 ± 1.07^a^	0.98 ± 0.22^d^	4.87 ± 0.50^b^	2.13 ± 0.15^c^	2.73 ± 0.40^c^	0.0001
Intromission Frequency*	59.51 ± 4.70^d^	203.90 ± 15.78^a^	132.03 ± 11.32^c^	173.71 ± 14.02^b^	169.70 ± 18.41^b^	0.0001

Control, no treatment; Sildenafil, rats treated with Sildenafil (5 mg/kg/d); Mucuna, rats treated with Mucuna pruriens (300 mg/kg bw); Tribulus; rats treated with Tribulus terrestris (300 mg/kg bw); Ashwagandha; rats treated with Ashwagandha (300 mg/kg bw). Data are LS means ± SE (*n* = 7). Different superscripts in the same row (a–c) indicate group mean differences (*p* < 0.05). * The number of intromissions in an ejaculatory series

The effects of extracts on sperm motility, sperm count, and abnormal sperm rate were shown in Table 3. Sperm motility was significantly different between the treatment groups (*P* < 0.05). Rats that were supplemented with *Mucuna and Tribulus* showed higher mean value for sperm motility compared with the control groups as shown in Table 3. However, *Tribulus* group showed the highest total sperm motility percentage than the other groups with a considerably high mean level of 84.29 % (*P* < 0.05). *Mucuna*, *Tribulus*, and Ashwagandha supplementation also produced a significant increase in sperm counts compared to the control group (*P* < 0.05). *Tribulus* group indicated the highest number of sperm count with a mean level of 161.42 million/right cauda epididymis (*P* < 0.05). However, there was no statistically significant difference in head, tail and total abnormal sperm rates (*P* > 0.05).

**Table 3:** The effects of extracts on sperm characteristics and abnormal sperm rate (%) in rats

Item	Groups					**--** *P* **--**
	Control	Sildenafil	*Mucuna pruriens*	*Tribulus terrestris*	*Ashwagandha*	
Total Motility,%	75.00 ± 5.49^bc^	80.00 ± 4.16^b^	80.00 ± 4.16^b^	84.29 ± 5.34^a^	78.00 ± 5.37^b^	<0.05
Count *	110.33 ± 37.78^b^	146.29 ± 21.55^a^	148.57 ± 31.45^a^	161.42 ± 36.11^a^	154.80 ± 10.55^a^	<0.05
Abnormal sperm rate, %						
Head	5.00 ± 3.85	3.71 ± 2.56	4.43 ± 2.76	3.00 ± 1.53	5.40 ± 2.88	>0.05
Tail	5.17 ± 2.14	4.86 ± 1.35	5.00 ± 2.45	3.86 ± 1.68	7.40 ± 3.21	>0.05
Total	10.17 ± 4.71	8.57 ± 2.37	9.43 ± 3.82	6.86 ± 2.11	12.80 ± 5.31	>0.05

Control, no treatment; Sildenafil, rats treated with Sildenafil (5 mg/kg/d); Mucuna, rats treated with *Mucuna pruriens* (300 mg/kg bw); Tribulus; rats treated with *Tribulus terrestris* (300 mg/kg bw); Ashwagandha; rats treated with Ashwagandha (300 mg/kg bw). Data are LS means ± SE (*n* = 7). Different superscripts in the same row (a–c) indicate group mean differences (*p* < 0.05). *Million/right cauda epididymis

The hematological data of the groups was presented in Table 4. No statistical significance was detected among all blood parameters of the Ashwagandha, *Tribulus* and *Mucuna* plant extract groups when compared to positive control sildenafil group and negative control standard group (*P* > 0.05). The effects of the extracts on serum biochemical parameters were shown in Table 5. There was no significant difference among the AST, ALT, CK, urea and creatine levels between the groups (*P* > 0.05), despite serum ALP level of *Mucuna* group was lower than the other groups (*P* < 0.05).

**Table 4:** The effects of extracts on blood parameters in rats

Item	Groups					--*P*--
	Control	Sildenafil	*Mucuna pruriens*	*Tribulus terrestris*	*Ashwagandha*	
L WBC	8.59 ± 0.31	8.73 ± 0.34	8.66 ± 0.45	8.98 ± 0.68	8.59 ± 0.22	>0.05
LYM, %	62.03 ± 7.18	62.53 ± 5.90	60.98 ± 12.69	61.19 ± 9.99	64.58 ± 4.47	>0.05
MID, %	14.07 ± 4.37	12.31 ± 3.85	12.20 ± 2.63	16.30 ± 6.67	11.82 ± 4.39	>0.05
GRAN, %	23.90 ± 5.26	25.16 ± 6.01	26.81 ± 13.67	22.51 ± 3.99	23.60 ± 3.10	>0.05
MID, # 10*3/μl	6.62 ± 0.53	6.60 ± 0.52	6.53 ± 0.39	7.11 ± 0.94	6.35 ± 0.65	>0.05
GRAN, # 10*3/μl	7.13 ± 0.42	7.32 ± 0.39	7.26 ± 0.79	7.46 ± 0.87	7.16 ± 0.23	>0.05
RBC, 10*6/μl	15.96 ± 0.05	15.96 ± 0.5	16.00 ± 0.06	15.96 ± 0.07	15.99 ± 0.04	>0.05
HGB, g/dL	16.27 ± 0.96	16.14 ± 0.65	16.57 ± 0.81	16.14 ± 1.06	16.60 ± 0.67	>0.05
HCT, %	81.27 ± 5.63	79.93 ± 3.51	81.63 ± 3.62	80.64 ± 5.79	81.16 ± 2.78	>0.05
MCV, fL	94.75 ± 2.56	94.13 ± 5.61	91.94 ± 4.42	94.69 ± 2.81	91.90 ± 1.76	>0.05
MCH, pg	18.90 ± 0.37	18.94 ± 0.88	18.29 ± 0.56	18.89 ± 0.38	18.72 ± 0.50	>0.05
MCHC, d/dL	19.97 ± 0.60	20.14 ± 0.40	20.24 ± 0.51	19.56 ± 1.38	20.42 ± 0.33	>0.05
RDW-CV, %	17.32 ± 0.58	16.47 ± 1.36	17.24 ± 1.19	17.90 ± 1.09	17.76 ± 0.83	>0.05
PLT, 10*3/μl	13.79 ± 0.11	13.60 ± 0.17	13.82 ± 0.20	13.76 ± 0.19	13.85 ± 0.20	>0.05
MPV, fL	11.62 ± 0.35	11.36 ± 0.46	11.86 ± 0.16	11.44 ± 0.40	11.68 ± 0.22	>0.05
PDW, %	17.53 ± 1.31	16.99 ± 1.09	16.74 ± 1.36	16.83 ± 0.94	16.20 ± 0.91	>0.05
PCT, %	1.14 ± 0.13	0.92 ± 0.15	1.22 ± 0.28	1.09 ± 0.24	1.22 ± 0.21	>0.05

*LWBC* leukocyte white blood cells, *LYM* lymphocyte, *MID* minimum inhibitory dilution, *GRAN* granulocyte, *RBC* red blood cell, *HGB* hemoglobin, *HCT* hematocrit, *MCV* mean corpuscular volume, *MCH* mean corpuscular hemoglobin, *MCHC* mean corpuscular hemoglobin concentration, *RDW-CV* red cell distribution width- coefficient variation, *PLT* platelet, *MPV* mean platelet volume, *PDW* platelet distribution width, *PCT* platelet crit; Control, no treatment; Sildenafil, rats treated with Sildenafil (5 mg/kg/d); Mucuna, rats treated with Mucuna pruriens (300 mg/kg bw); Tribulus; rats treated with Tribulus terrestris (300 mg/kg bw); Ashwagandha; rats treated with Ashwagandha (300 mg/kg bw). Data are LS means ± SE (*n* = 7). Different superscripts in the same row (a–c) indicate group mean differences (*p* < 0.05)

**Table 5:** The effects of extracts on serum biochemical parameters in rats

Item	Groups					--*P*--
	Control	Sildenafil	*Mucuna pruriens*	*Tribulus terrestris*	*Ashwagandha*	
AST, U/L	97.50 ± 8.92	118.86 ± 33.73	99.29 ± 15.85	104.29 ± 21.86	108.51 ± 23.25	>0.05
ALT, U/L	46.67 ± 6.50	59.86 ± 10.48	48.71 ± 6.78	47.71 ± 10.24	50.00 ± 10.05	>0.05
ALP, U/L	204.00 ± 43.32^a^	202.14 ± 31.02^a^	161.86 ± 29.29^b^	215.86 ± 25.58^a^	202.00 ± 22.03^a^	0.0349
CK, U/L	531.00 ± 224.85	399.29 ± 292.28	405.86 ± 189.07	226.86 ± 102.12	331.40 ± 44.57	>0.05
Urea, mg/dl	33.00 ± 2.61	36.43 ± 2.07	36.14 ± 5.08	36.00 ± .3.74	36.80 ± 6.18	>0.05
Creatine, mg/dl	0.30 ± 0.03	0.28 ± 0.03	0.28 ± 0.04	0.29 ± 0.02	0.30 ± 0.03	>0.05

*AST* aspartate aminotransferase, *ALT* alanine aminotransferase, *ALP* alkaline phosphatase, *CK* creatine kinase control, no treatment; Sildenafil, rats treated with Sildenafil (5 mg/kg/d); Mucuna, rats treated with Mucuna pruriens (300 mg/kg bw); Tribulus; rats treated with Tribulus terrestris (300 mg/kg bw); Ashwagandha; rats treated with Ashwagandha (300 mg/kg bw). Data are LS means ± SE (*n* = 7). Different superscripts in the same row (a–c) indicate group mean differences (*p* < 0.05)

When we examined the effects of extracts on serum hormones and MDA (Table 6), FSH and LH hormone levels showed no significance (*P* > 0.05) but serum testosterone levels were 1.84, 1.76 and 1.58 fold higher than the normal control group in Sildenafil, *Tribulus* and Ashwagandha groups respectively (*P* < 0.0001). *Mucuna pruriens* administration did not show significance on serum testosterone levels when compared to standard control group of rats (*P* > 0.05). All of the extract treatments to the groups lowered the serum and testis tissue MDA levels significantly, in comparison to sildenafil group of rats (*P* < 0.0001). The most prominent ameliorating decrease was in *Tribulus* group with a 32 % in serum and 14 % in testis MDA levels (*P* < 0.0001).

**Table 6:** The effects of extracts on serum hormones and MDA levels in rats

Item	Groups					--*P*--
	Control	Sildenafil	*Mucuna pruriens*	*Tribulus terrestris*	*Ashwagandha*	
FSH, mIU/ml	0.33 ± 0.22	0.40 ± 0.37	0.31 ± 0.25	0.34 ± 0.39	0.31 ± 0.24	>0.05
LH, mIU/ml	0.22 ± 0.29	0.30 ± 0.23	0.25 ± 0.17	0.24 ± 0.19	0.27 ± 0.19	>0.05
Testosterone, ng/ml	2.27 ± 0.17^c^	4.14 ± 0.71^a^	2.57 ± 0.44^c^	3.99 ± 0.16^ab^	3.58 ± 0.18^b^	0.0001
Serum MDA, μmol/L	0.54 ± 0.05^a^	0.56 ± 0.06^a^	0.49 ± 0.04^b^	0.38 ± 0.02^c^	0.44 ± 0.02^b^	0.0001
Testis MDA, nmol/g	1.64 ± 0.09^ab^	1.68 ± 0.09^a^	1.55 ± 0.06^cb^	1.45 ± 0.09^d^	1.51 ± 0.07^cd^	0.0001

*FSH* follicle-stimulating hormone, *LH* luteinizing hormone, *MDA* malonaldeyhde; Control, no treatment; Sildenafil, rats treated with Sildenafil (5 mg/kg/d); Mucuna, rats treated with Mucuna pruriens (300 mg/kg bw); Tribulus; rats treated with Tribulus terrestris (300 mg/kg bw); Ashwagandha; rats treated with Ashwagandha (300 mg/kg bw). Data are LS means ± SE (*n* = 7). Different superscripts in the same row (a–c) indicate group mean differences (*p* < 0.05)

**Fig. 1:**
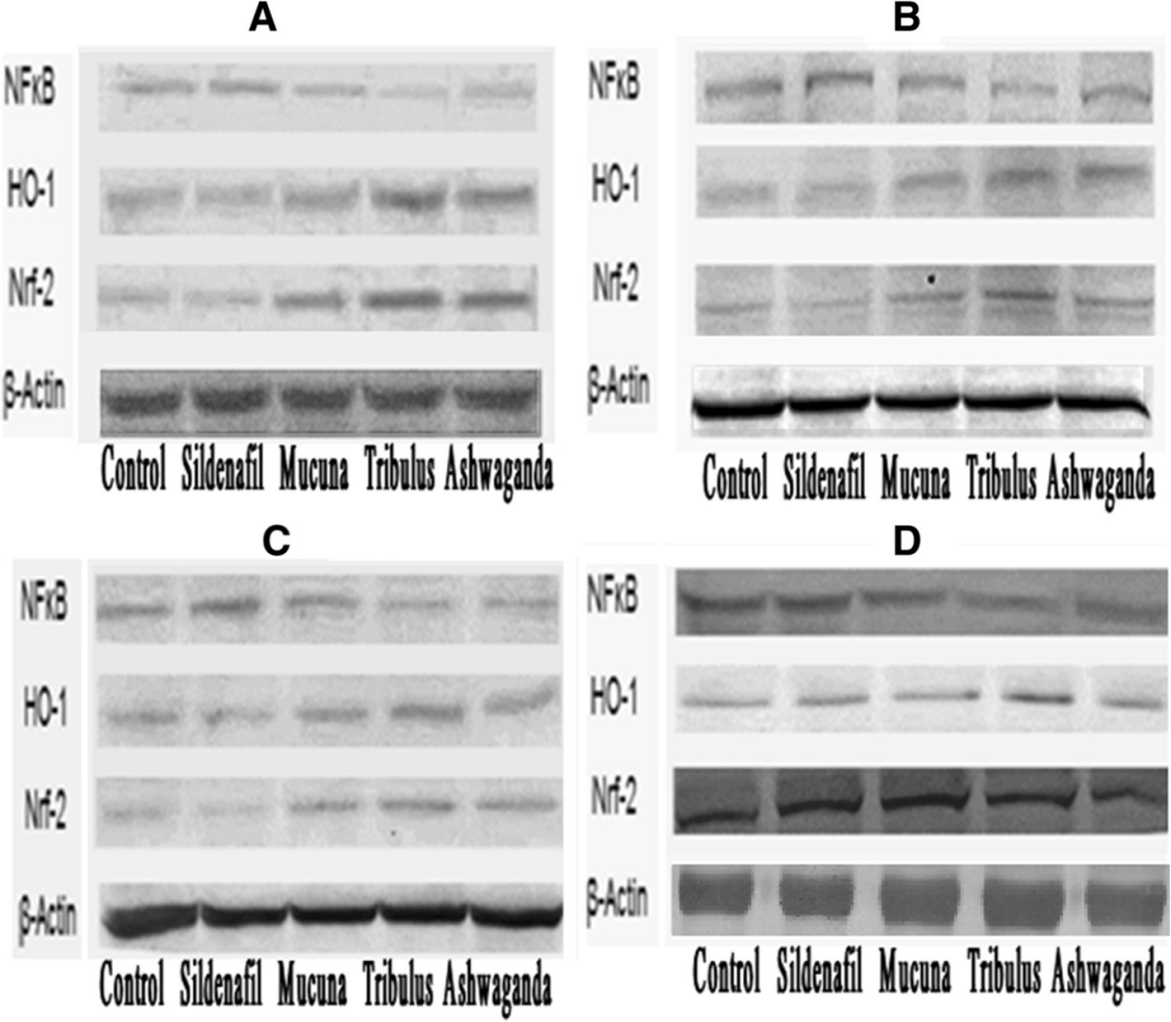
Epididymis tissue NF-κB, Nrf-2 and HO-1 levels (**a**); prostate tissue NF-κB, Nrf-2 and HO-1 levels (**b**); testes tissue NF-κB, Nrf-2 and HO-1 levels (**c**); vas deferens tissue NF-κB, Nrf-2 and HO-1 levels (**d**) western blot bands. Data are expressed as a ratio of normal control value (set to 100 %). Blots were repeated at least 4 times (*n* = 4) and a representative blot is shown. Actin was included to ensure equal protein loading. The bars represent the standard error of the mean. Data points with different superscripts are significantly different at the level of *P* < 0.05 by Fisher’s multiple comparison test

**Fig. 2:**
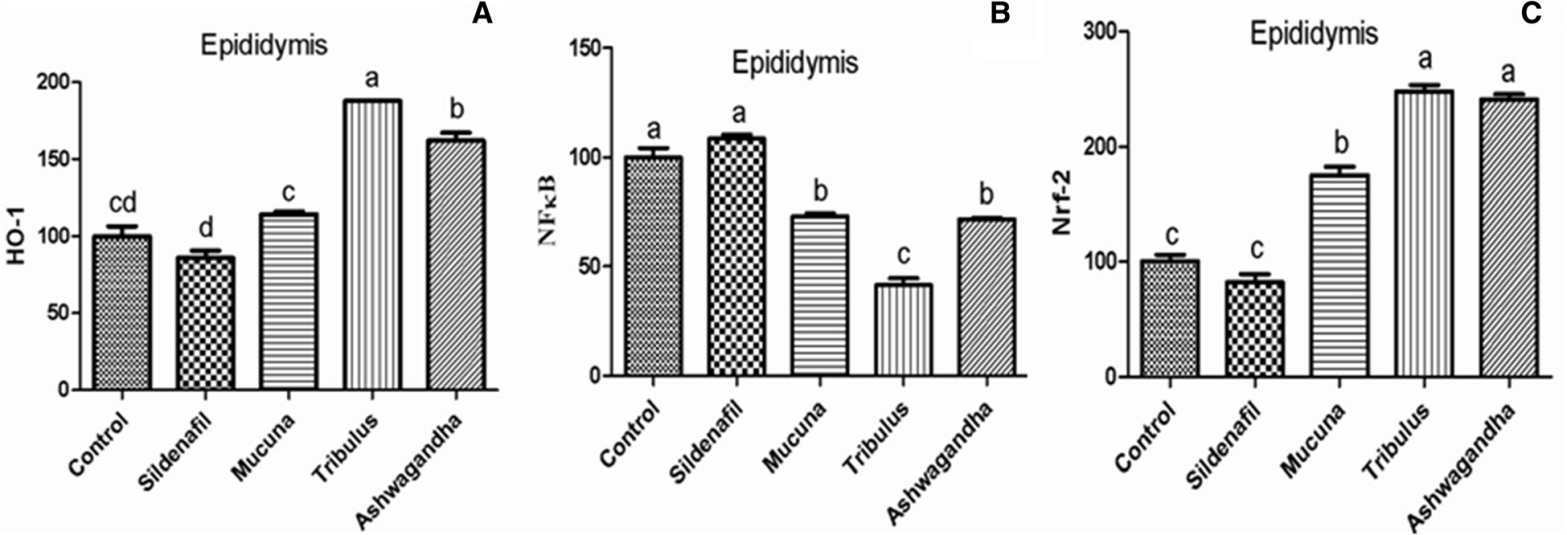
HO-1 (**a**), NF-κB (**b**) and Nrf-2 (**c**) Protein levels of epididymis tissue in male rats (*P* < 0.0001). Data are expressed as a ratio of normal control value (set to 100 %). Blots were repeated at least 4 times (*n* = 4) and a representative blot is shown. Actin was included to ensure equal protein loading. The bars represent the standard error of the mean. Data points with different superscripts are significantly different at the level of *P* < 0.05 by Fisher’s multiple comparison test

**Fig. 3:**
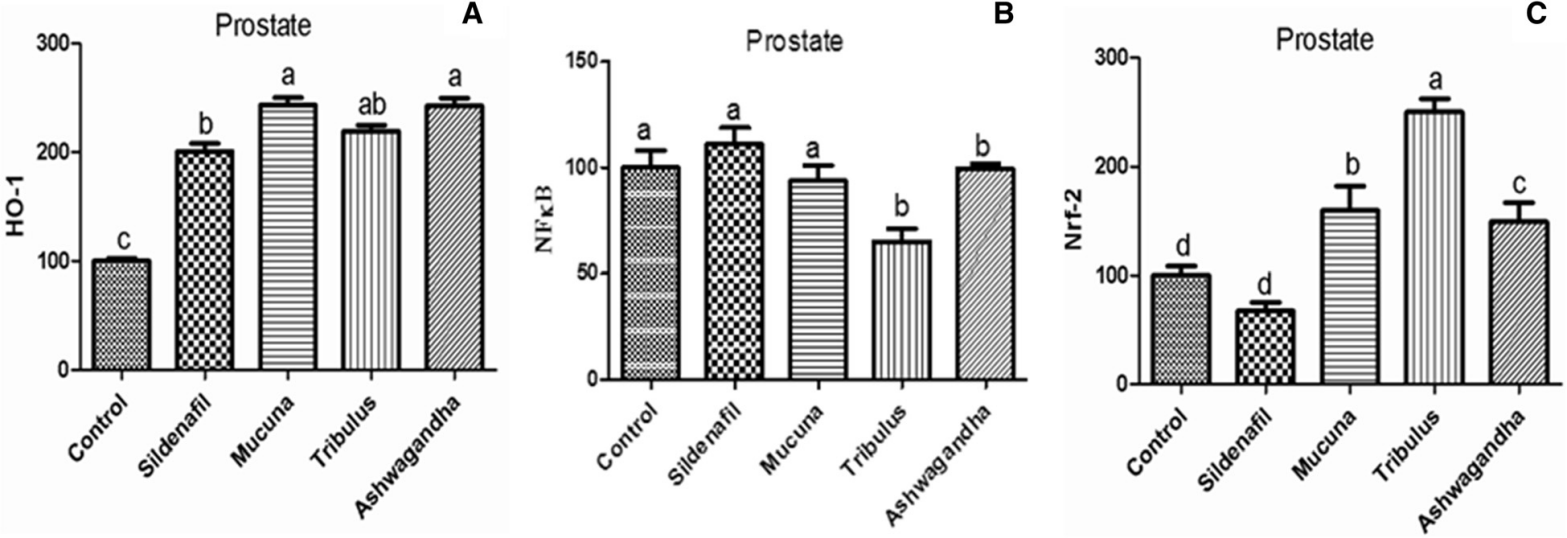
HO-1 (**a**), NF-κB (**b**) and Nrf-2 (**c**) Protein levels of prostate tissue in male rats (*P* < 0.0001). Data are expressed as a ratio of normal control value (set to 100 %). Blots were repeated at least 4 times (*n* = 4) and a representative blot is shown. Actin was included to ensure equal protein loading. The bars represent the standard error of the mean. Data points with different superscripts are significantly

**Fig. 4:**
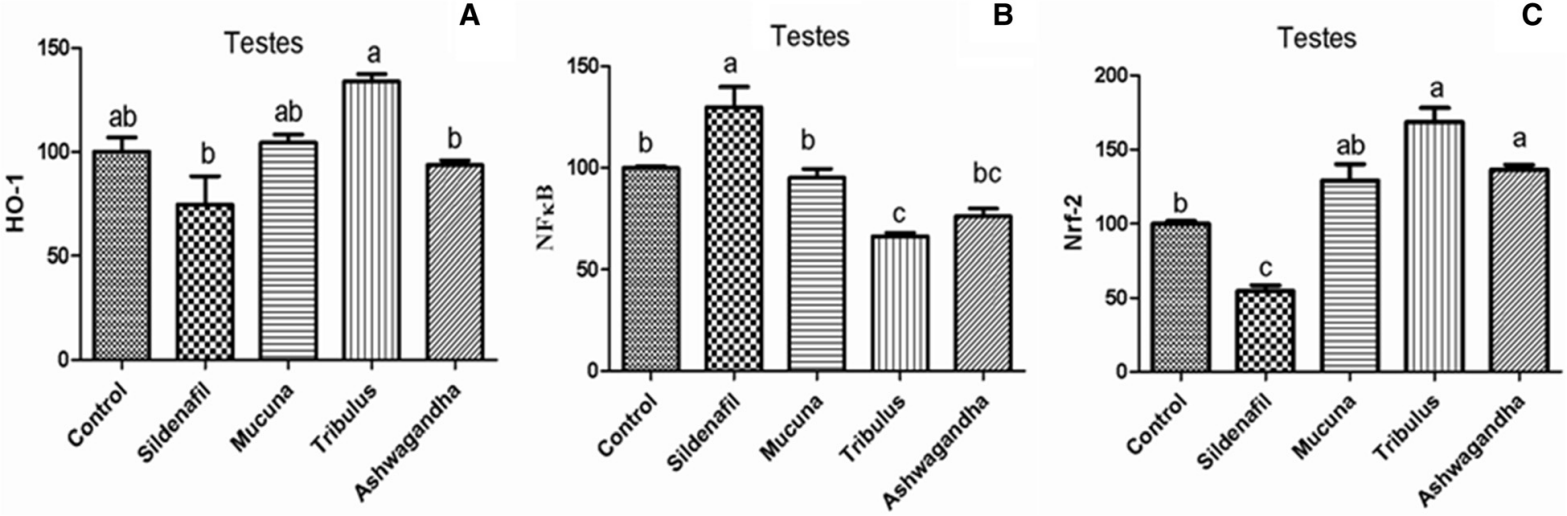
HO-1 (**a**), NF-κB (**b**) and Nrf-2 (**c**) levels of testes tissue in male rats (*P* < 0.0001). Data are expressed as a ratio of normal control value (set to 100 %). Blots were repeated at least 4 times (*n* = 4) and a representative blot is shown. Actin was included to ensure equal protein loading. The bars represent the standard error of the mean. Data points with different superscripts are significantly different at the level of *P* < 0.05 by Fisher’s multiple comparison test

**Fig. 5:**
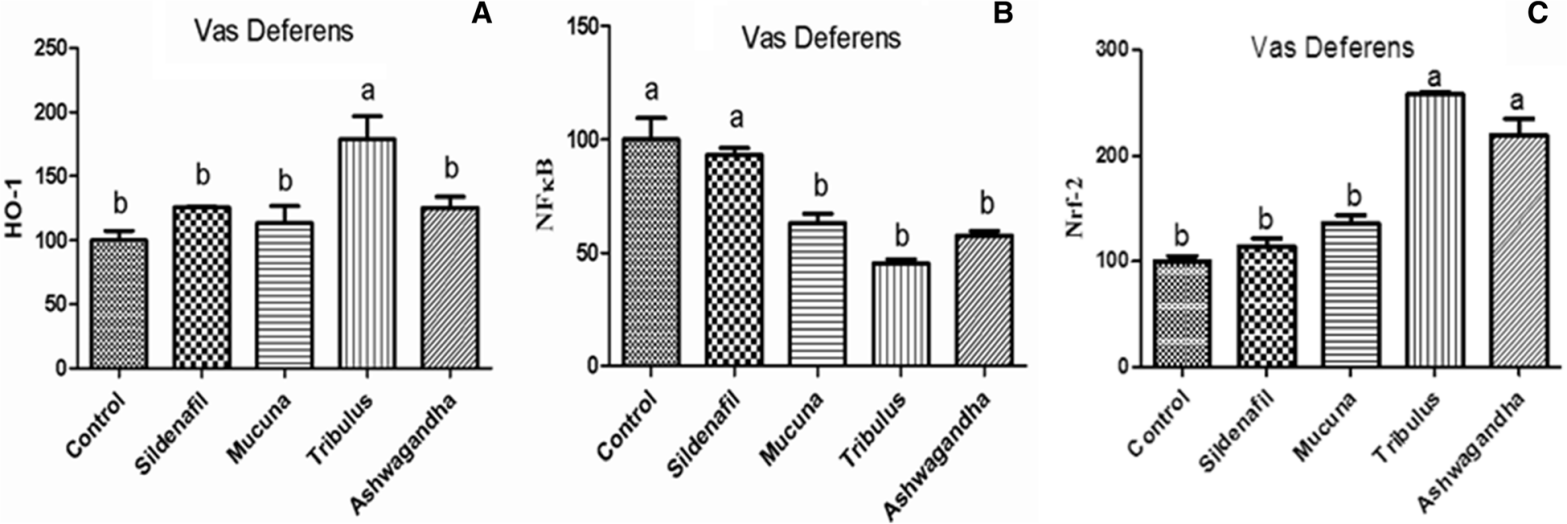
HO-1 (**a**), NF-κB (**b**) and Nrf-2 (**c**) Protein levels of vas deferens tissue in male rats (*P* < 0.0001). Data are expressed as a ratio of normal control value (set to 100 %). Blots were repeated at least 4 times (*n* = 4) and a representative blot is shown. Actin was included to ensure equal protein loading. The bars represent the standard error of the mean. Data points with different superscripts are significantly different at the level of *P* < 0.05 by Fisher’s multiple comparison test

Figure 1(a–d), shows the western blot bands of the Control, Sildenafil, *Mucuna*, *Tribulus* and Ashwagandha plant extract groups on the expression levels of NF-κB and HO-1/Nrf2 in reproductive tissues which included epididymis, prostate, testes and vas deferens. In the epididymis tissue (Fig. 2a–c), higher levels of HO-1/Nrf2 were found in *Tribulus*, *Mucuna* and Ashwagandha groups compared to the other groups, in contrast, NF-κB levels were significantly lower in *Tribulus*, *Mucuna* and Ashwagandha administered groups (*P* < 0.0001). In the prostate tissue, HO-1/Nrf2 levels of *Tribulus*, *Mucuna*, and Ashwagandha extract treated groups significantly increased compared to the negative and positive controls while NF-κB levels decreased considerably in the same groups compared to the both controls (Fig. 3a–c), (*P* < 0.0001). *Tribulus* and *Mucuna* extract treated groups showed higher HO-1 levels in testis tissue than the other groups and also *Tribulus*, *Mucuna* and Ashwagandha groups Nrf2 levels were significantly higher than Sildenafil and Standard control (Fig. 4a,c), (*P* < 0.0001). Furthermore, the least NF-κB expression level was found in *Tribulus* group in the testes and also *Mucuna* and Ashwagandha groups suggested a significant decrease in comparison to positive control Sildenafil group (Fig. 4b) (*P* < 0.0001). In vas deferens tissue HO-1 expression, *Tribulus* extract treated rats showed the highest levels among the groups (Fig. 5a) (*P* < 0.0001), while significantly higher Nrf2 levels, were again found in *Tribulus* and moreover Ashwagandha groups (Fig. 5c) (*P* < 0.0001). All herbal extract treated groups showed significantly lower NF-κB levels when compared to both positive and negative controls (Fig. 5b) (*P* < 0.0001).

**Fig. 6:**
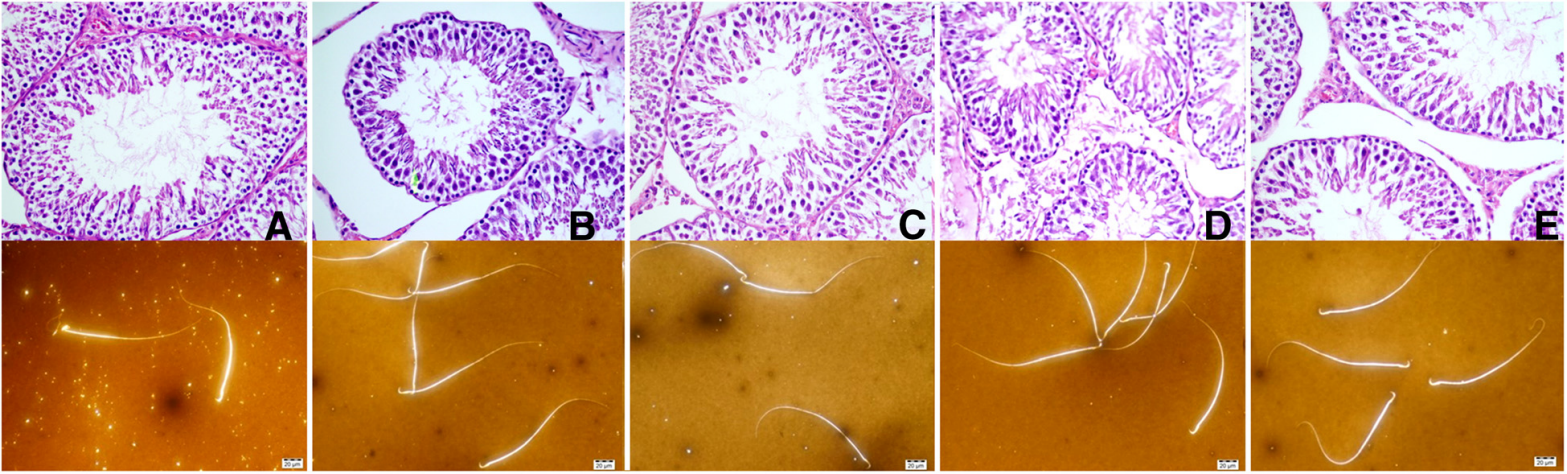
Representative photomicrographs of histopathological structure of testes and below sperm morphologies for each in different treatment groups. **a**: Control, no treatment; **b**: Sildenafil, rats treated with sildenafil (5 mg/kg/d); **c**: Mucuna, rats treated with *Mucuna pruriens* (300 mg/kg bw); **d**: Tribulus; rats treated with *Tribulus terrestris* (300 mg/kg bw); **e**: Ashwagandha; rats treated with Ashwagandha (300 mg/kg bw)

No significant change was observed neither in testes histopathology nor the sperm morphologies between the groups as shown in (Fig. 6a–e).

## Discussion

Male infertility, erectile dysfunction, and reproductive system problems are common public health disorders besides a stressed out way of life has been increasing the sexual dysfunction suffering subjects around the world [2, 35]. A better understanding of erectile and sexual functions at molecular levels in the male reproductive system shall be a great achievement for the aim of precise aphrodisiac substance choice [35]. In this study, we tried to figure out the sexual enhancing capacity, blood and serum biochemical parameters and hormones, antioxidant capacity by assessing the serum and testis MDA and Nrf2 pathway of reproductive organ parts of the male rats by feeding the animals with *Tribulus terrestris*, *Mucuna pruriens* and Ashwagandha extracts and comparing them with standard control group and sildenafil-treated group as a positive control.

*Mucuna pruriens* is a traditional Ayurvedic Indian medicinal plant and has been used in Indian medicine for a long time. The total alkaloids from *Mucuna* seeds were reported to increase sperm production and weight of some reproductive organ parts in the albino rats [36]. In our study, we also found out an increase of the sperm production by the effect of the *Mucuna* and the other extracts but there was no significant change observed in any reproductive tissue weights according to our results. In many studies, *Mucuna pruriens* seed powder were observed to significantly and sustainably improve sexual behaviors such as; increased mounting frequency and intromission frequency and decreased mounting latency and intromission latency as parallel to our results [37–39]. However, *Mucuna pruriens* hydrolysates were shown to be hypocholesterolemic and hypolipidemic effective and rich in protein content [40]. Diminishing the ROS level, MMP renewal, and apoptosis regulation through *Mucuna pruriens* improved the spermatogenesis mainly via its major component L-DOPA was shown in previous studies [41, 42], similarly as our study showed decreasing MDA levels, NF-κB protein expression, and increasing HO-1/NrF-2 levels correspondingly. *Mucuna* administration increased testosterone and LH levels and decreased lipid peroxidation and FSH in infertile men also stimulated the antioxidant enzymes and hormones such as catalase, SOD and GSH via reactivating the antioxidant defense system and recovered sperm count and motility [43–45]. In parallel to previous results, *Mucuna pruriens* treatment improved testosterone levels and significantly recovered sperm count and motility also lowered lipid peroxidation significantly but did not alter the FSH and LH levels in our study.

*Tribulus terrestris* is regarded to be an aphrodisiac herb and has been used in traditional Far East medicine for ages because of its reputation to improve sexual functions along with its beneficial effects on various diseases [21, 22, 35, 46]. It was shown that *Tribulus terrestris* administration improved LH also sperm production and testosterone levels in rams [47] as we consistently showed its enhancing effect on sperm counts and testosterone levels except LH levels were determined indifferent than the control. According to our study, *Tribulus* group showed significant improvements in sexual behaviors, including; increased mounting and intromission frequencies and decreased mounting and intromission latencies similar to previously reported studies [7, 21, 48]. *Tribulus terrestris* were shown to increase the nitric oxide release and this is considered for its aphrodisiac capacity character [48, 49]. Our results suggested major increases in Nrf-2 and HO-1 levels and decreases in NF-κB and MDA levels of the various reproductive tissue parts and serum when administered with *Tribulus terrestris* similarly to a study which the extract was restored antioxidant enzyme activity and their expression profile in kidney tissue [50] and another study that extract blocked proliferation and induced apoptosis in cancer cells through the inhibition of NF-κB signaling [51].

Ashwagandha (*Withania somnifera*) has been known as Indian Ginseng and regarded as adaptogen, tonic with its aphrodisiac properties [35]. Even though an old study reported that it was shown as antifertility effective and mating behavior reducer in mice [52], recent studies reported its capability of combating stress-induced infertility and its protective effect to some reproductive endocrine dysfunctions in male rats were shown [53, 54]. Our study has been suggested Ashwagandha be effective as a sexual enhancer not as satisfactory as *Tribulus terrestris* but reasonably adequate.

## Conclusions

The present study provides evidence that the extracts of *Tribulus*, Ashwagandha and *Mucuna* are potent enhancers of sexual function and behavior by the increasing testosterone levels and regulation of NF-κB and Nrf2/HO–1 pathways in male rats. The results of the present study have also indicated that *Tribulus* extract was comparatively more potent than the corresponding Ashwagandha and *Mucuna* extracts for the sexual functions. Moreover, further studies should be carried out to check the molecular markers related to the sexual function in male rats.

## Abbreviations

ALP: Alkaline phosphatase
ALT: Alanine aminotransferase
ARE: Antioxidant response element
AST: Aspartate aminotransferase
CK: Creatine kinase
FSH: Follicle-stimulating hormone
GRAN: Granulocyte
HCT: Hematocrit
HGB: Hemoglobin
HO-1: Heme oxygenase-1
LH: Luteinizing hormone
LWBC: Leukocyte white blood cells
LYM: Lymphocyte
MCH: Mean corpuscular hemoglobin
MCHC: Mean corpuscular hemoglobin concentration
MCV: Mean corpuscular volume
MDA: Malondialdehyde
MID: Minimum inhibitory dilution
MPV: Mean platelet volume
NF-κB: Nuclear factor kappa B
NRF2: Erythroid 2-related factor 2
PCT: Platelet crit
PDW: Platelet distribution width
PLT: Platelet
RBC: Red blood cell
RDW-CV: Red cell distribution width-coefficient variation
